# Reliability of the Aboriginal Children’s Health and Well-Being Measure (ACHWM)

**DOI:** 10.1186/s40064-016-3776-y

**Published:** 2016-12-07

**Authors:** Nancy L. Young, Mary Jo Wabano, Koyo Usuba, Debbie Mishibinijima, Diane Jacko, Tricia A. Burke

**Affiliations:** 1 Laurentian University, 935 Ramsey Lake Road, Sudbury, ON Canada; 2 Naandwechige Gamig Wiikwemkoong Health Centre, Wiikwemkoong, ON Canada; 3 Nadmadwin Mental Health, Wiikwemkoong, ON Canada

**Keywords:** Questionnaire, Reliability, Indigenous peoples, Children and youth

## Abstract

**Purpose:**

The aim of this research was to evaluate the reliability of the Aboriginal Children’s Health and Well-Being Measure© (ACHWM).

**Methods:**

Two cohorts of children from Wiikwemkoong Unceded Territory were recruited for this study. Each child completed the ACHWM independently on a computer tablet running a customized survey app. The data from the first and second cohorts were used to estimate the internal consistencies using Cronbach’s alpha. A subgroup of the second cohort completed the survey twice, within the same day. The data from this subgroup was used to evaluate the test–retest reliability using a random effects Intra-class Correlation Coefficient (ICC).

**Results:**

There were 124 participants in the first cohort and 132 participants in the second cohort. The repeated measures subgroup was comprised of 29 participants from the second cohort. The internal consistency statistic (Cronbach’s alpha) was 0.93 for the first and second cohorts. The test–retest reliability ICC was 0.94 (95% CI 0.86–0.97) for the ACHWM summary scores based on the repeated measures subgroup.

**Conclusions:**

These results establish the internal consistency and the test–retest validity of the ACHWM. This important finding will enable Aboriginal communities to use this measure with confidence and promote the voices of their children in reporting their health. The ACHWM is an essential data gathering tool that enables evidence-based health care for Aboriginal communities.

## Background

The Aboriginal Children’s Health and Well-Being Measure (ACHWM) is a self-report survey that was developed to assess the holistic health of Aboriginal children and evaluate improvements over time. The 62 items within this measure were developed from the perspectives of Aboriginal children, through 6 full-day focus groups involving 34 children between 8 and 18 years of age, in Wiikwemkoong, Ontario, Canada (Young et al. 2013). The ACHWM development was conceptually-driven, based on the Medicine Wheel Framework (Dumont 2005). Thus, it includes questions to assess each of the 4 quadrants (or domains) of health: spiritual, emotional, physical and mental. Note that based on this framework, the mental quadrant is about thinking and problem solving (cognition) whereas feelings were reflected in the emotional quadrant.

As part of the development of a new measure, it is well established in the literature that certain core metrics must be evaluated for new patient-reported outcomes. These metrics include the: conceptual foundation; validity, reliability, responsiveness, interpretability, respondent and administrative burden, alternative forms, and cultural and language adaptations (translations) (Terwee et al. 2007; Aaronson et al. 2002). The conceptual foundation was presented in the initial development paper on the ACHWM (Young et al. 2013). The validity of the ACHWM was assessed in comparison to the PedsQL (ACHWM mean = 71.4; PedsQL mean = 71.1; r = 0.52 p = 0.0001) based on data from 48 participants in a community survey in one First Nation community in 2014 (Young et al. 2015). The validity was replicated in another First Nation in comparison to the KidsScreen (n = 25; ACHWM 78.8; Kidscreen 76.8; r = 0.63 p = 0.0007) in 2015 (Kristensen-Didur et al. 2015). Five Aboriginal communities in Canada are currently utilizing this measure and many others have expressed interest, based on its development model and cultural applicability. It is essential that the other measurement properties be established in the literature so that communities and researchers can make evidence-informed decisions.

The purpose of this paper is to establish the internal consistency and test–retest reliability of the ACHWM.

## Methods

First Nations children living on the Wiikwemkoong Unceded Territory^1^ were invited to participate in a program of research that included annual community health surveys in 2014 and 2015, and a repeated measures study that was incorporated into the 2015 community survey. The latter included a sub-set of children who were invited to complete the survey twice. We estimated the required sample size to be approximately 30 children based on an expected intra-class correlation of >0.8 with 2 time points (Donner and Eliasziw 1987).

Children were asked to complete the ACHWM independently using a computer tablet. The tablet version was critical to enable those with low literacy levels to participate via a text-to-speech option. It also enhanced feasibility via direct data entry. All android tablets used store-and-forward technology to accommodate community resources. Data were stored on a REDCap database (Harris et al. 2009) on a server housed at Laurentian University.

### Samples and data collection

Two cohorts of children (8–18 years of age) from Wiikwemkoong were recruited for this study. Each child completed the ACHWM independently on a computer tablet running a customized survey app. The first cohort completed the ACHWM between August 2013 and March 2014 as part of a community-based child health survey. Data from the first sample was used to estimate the internal consistency of the items within the summary score, using Cronbach’s alpha. The second cohort completed the ACHWM between August 2014 and February 2015 as part of the second annual community-based health survey. Data from the second cohort was used to confirm the internal consistency, based on new data. Note that the 58-item version ACHWM was the current standard at the time of first community survey. Four additional questions were added at the beginning of the second community survey and were treated as missing data in the first cohort.

A subgroup of the children in the second cohort completed the ACHWM twice, within the same day, in April 2015. The selection of the time interval was an important consideration in the design of this study. We began by considering the standard interval of 2 weeks with the goal of minimizing recall bias, and at the same time reducing the chance that true change in the construct has taken place (Streiner and Norman 2008; McDowell and Newell 1987). However, in pilot testing we found that very few of the children returned 2 weeks later, and of those who returned, many had experienced changes in their lives which could impact their ACHWM scores. Thus, we elected to conduct both tests on the same day, with a variety of activities in between. In addition, the survey was presented on a tablet computer one item at a time, making it difficult to remember patterns of responses. The completion of the survey twice on the same day is a method that has been previously established in populations that are expected to change quickly (Paiva et al. 2014). Furthermore, Marx et al. (2003) studied the relationship between days between measures and concordance statistics and found equivalence between 2 and 14 days.

### Analyses

The analysis began by describing the study cohorts in terms of the sample size, age, gender, self-rated health and ACHWM scores. The internal consistency of the items within each of the quadrants was assessed using Cronbach’s alpha (Bland and Altman 1997) for each cohort separately, to determine the stability of the estimates. The level of acceptability for Cronbach’s alpha was specified a priori as 0.7 based on the literature (Bland and Altman 1997).

Data from the test–retest subgroup within the second cohort was used to determine the repeated measures test–retest reliability of the ACHWM. Test–retest reliability was assessed using a random-effects Intra-class Correlation Coefficient (ICC) to assess concordance (Shrout and Fleiss 1979; Fleiss 1986). The ICC values were interpreted as: excellent >0.75, fair to good 0.40–0.75, fair/poor <0.40 based on the literature (Fleiss et al. 2013).

### Ethics approvals

This project was approved by: Wiikwemkoong Chief and Council (BCMs #418-2013 and #632-2014); the Laurentian University Research Ethics Board (2012-11-17); and the Manitoulin Anishinaabek Research Review Committee (Feb 3, 2013). Written informed consent was obtained from parents and children. This consent allows for publication of aggregate data. The raw data for this research are not publicly available, due to the ethical requirement to ensure confidentiality.

## Results

The Wiikwemkoong community has successfully completed two annual community surveys: 2014 and 2015. The reliability analyses were conducted using all available data from these two annual surveys. Thus we were able to include the 124 participants from the first annual community survey and 132 participants from the second annual community survey, with a subgroup of 29 participants in the second annual survey completing the ACHWM twice. Thus, the combined sample for the reliability analyses was 256 children. The sample characteristics for each of the cohorts are presented in Table 1.

**Table 1:** Sample characteristics

Cohorts	Purpose	Number of participants	Age Mean (SD) [range]	Gender	Excellent or very good health	ACHWM Mean (SD) [range]
First	Evaluate internal consistency	124	14.5 (3.9) [7.6–21.7]	69 girls (56%)	38.3%	72.5 (11.6) [40.3–98.7]
Second	Confirm internal consistency	132	13.2 (3.4) [8.2–21.8]	69 girls (56%)	47.0%	75.1 (11.7) [39.0–98.8]
	Determine test–retest reliability	29	16.8 (3.2) [8.9–21.8]	14 girls (48%)	58.6%	78.8 (9.2) [57.5–96.0]

### Internal consistency

The internal consistency for the ACHWM summary score was 0.93 for both 2014 and 2015 cohorts, indicating that the ACHWM has excellent internal consistency. Furthermore, this result meets the standards set by Bland and Altman (1997) for use with individual patients in clinical contexts. The internal consistency results for the ACHWM summary score and quadrant scores are presented for both cohorts in Table 2. Three of the quadrant scores met the criteria we established a priori. The fourth quadrant (mental) approached the standard but did not meet it. Note that the mental quadrant is focused on thinking and problem solving and has the fewest items (9).

**Table 2:** Cronbach’s alpha coefficients

	Sample size	Summary (62 items)	Spiritual (16 items)	Emotional (24 items)	Physical (13 items)	Mental (9 items)
First cohort	124	0.93	0.87	0.88	0.81	0.52
Second cohort	132	0.93	0.85	0.86	0.80	0.64

During the analysis of internal consistency of the summary and quadrant scores, we explored the relationship of items to their quadrant. Note that the allocation of items to quadrants was made during the ACHWM’s development, based on the expertise of children, using the Medicine Wheel framework as a guide (Young et al. 2013). This exploratory analysis confirmed the allocation of 57 items to their previously assigned quadrants. While there were 5 items that had a slightly better correlation with a different quadrant, the difference was very small and no change in allocation was recommended. This decision was also in keeping with respecting the children’s voices.

### Test–retest reliability

**Fig. 1:**
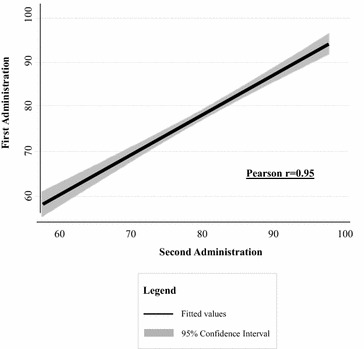
Correlation between test and re-test ACHWM scores

The repeated measures reliability was assessed using a test–retest approach evaluated with a random-effects ICC. The ACHWM summary score demonstrated concordance of 0.94 (95% CI 0.86–0.97) based on data from 29 participants in the repeated measures analysis. The correlation between the ACHWM summary scores from the first and second administrations are shown in Fig. 1.

The ICC estimates and confidence intervals for the ACHWM summary scores and each of the quadrant scores are presented in Table 3.

**Table 3:** Intra-class correlation coefficients

	Sample size	Summary (62 items)	Spiritual (16 items)	Emotional (24 items)	Physical (13 items)	Mental (9 items)
ICC (95% CI)	29	0.94 (0.86–0.97)	0.96 (0.91–0.98)	0.93 (0.83–0.97)	0.84 (0.62–0.93)	0.75 (0.53–0.87)

These results indicate that the ACHWM summary score and all quadrant scores met the criteria set by Fleiss et al. (2013) for excellent test–retest reliability.

## Discussion

This study reports on the reliability of a new self-report measure of health and wellness from the perspectives of Aboriginal children in Canada. The ACHWM is unique in that it was developed for and with Aboriginal children (Young et al. 2013, 2015a, b). The content validity of this measure has been established for First Nations, Métis and Inuit children in Canada, and it is being rapidly adopted by First Nations and Aboriginal agencies in Canada, and thus it is urgent that we establish its reliability.

The results of this study indicate that the internal consistency results for the overall summary score and three of the quadrant scores were excellent. The internal consistency results presented in Table 2 compare favourably with those reported by for the PedsQL, which ranged from 0.71 to 0.89 (Varni et al. 2003). Furthermore, the internal consistency of the ACHWM summary score exceeds the standard of 0.90 recommended for use with individual participant analysis (Nunnally and Bernstein 1994). The internal consistency was not as strong for the mental quadrant, which contained 9 items. The low number of items may be the source of the low alpha coefficients, and we are exploring opportunities to enhance the mental quadrant by the addition of items related to cognition. However, the results for the emotional quadrant, which is often referred to in other contexts as *“mental health”,* were very strong at 0.88 and 0.86. Overall, the internal consistency results support the allocation of items to quadrants that was made based on the expertise of children using the Medicine Wheel Framework, and later confirmed by an expert panel as described in the ACHWM development publication (Young et al. 2013).

The test–retest reliability of the ACHWM summary score was excellent and meets the requirement for use with individual participants. The test–retest reliability statistics for the quadrant scores were also very strong. These results indicate that Aboriginal children are consistent when reporting their health and well-being using the ACHWM.

The authors acknowledge that this study is based on a relatively small sample of children, and as a result confidence intervals are wide. In addition, the test–retest was conducted over a narrow time window, which was essential given the frequency of changes in their lives. Despite these limitations we have strong favourable results that are consistent with the literature for other child health metrics, such as the PedsQL, and thus support the use of this measure in the future.

## Conclusions

These results establish the internal consistency and the test–retest validity of the ACHWM and associated quadrant scores. These are important parameters that will enable Aboriginal communities across Canada to use this measure with confidence. Because the measure is based on child-self-report, it promotes the voices of children in understanding their health. The establishment of these key measurement properties is essential to enable evidence-based health care within Aboriginal communities. The ACHWM is now ready for use in evaluating the impact of various programs on Aboriginal children’s health.

The ACHWM also has potential relevance to Indigenous children in other regions of the world. It was been shared at the International Meeting on Indigenous Child Health in 2015 and will be offering a workshop at this meeting in 2017, to enable child health researchers and clinicians to examine its relevance in their local context. We have also developed and shared a detailed process for assessing the relevance of the ACHWM in other contexts (Young et al. 2013, 2015a, b). More information on the AHCWM can be found at http://www.ACHWM.ca.

## Footnotes

1 The official name of the First Nation was formerly Wikwemikong Unceded Indian Reserve, which has recently been changed to Wiikwemkoong Unceded Territory by its leadership.
